# Development and (pre-) clinical assessment of a novel surgical tool for primary and secondary tracheoesophageal puncture with immediate voice prosthesis insertion, the Provox Vega Puncture Set

**DOI:** 10.1007/s00405-012-1976-9

**Published:** 2012-03-06

**Authors:** Frans J. M. Hilgers, Kai J. Lorenz, Heinz Maier, Cees A. Meeuwis, Jeroen D. F. Kerrebijn, Vincent Vander Poorten, Anne Sophie Vinck, Miquel Quer, Michiel W. M. van den Brekel

**Affiliations:** 1 The Netherlands Cancer Institute, Plesmanlaan 121, 1066CX Amsterdam, The Netherlands; 2 Institute for Phonetic Sciences, and Academic Medical Center, University of Amsterdam, Amsterdam, The Netherlands; 3 German Armed Forces Hospital, Ulm, Germany; 4 Eramsus University Medical Center, Rotterdam, The Netherlands; 5 University Hospital Leuven, Leuven, Belgium; 6 Hospital de la Santa Creu i Sant Pau, Universitat Autònoma de Barcelona, Barcelona, Spain

**Keywords:** Total laryngectomy, Tracheoesophageal puncture (TEP), Voice prosthesis, Primary and secondary TEP, Seldinger technique

## Abstract

Development and (pre-) clinical assessment were performed of a novel surgical tool for primary and secondary tracheoesophageal puncture (TEP) with immediate voice prosthesis (VP) insertion in laryngectomized patients, the Provox Vega Puncture Set (PVPS). After preclinical assessment in fresh frozen cadavers, a multicenter prospective clinical feasibility study in two stages was performed. Stage-1 included 20 patients, and stage-2 had 27. Based on observations in stage-1, the PVPS was re-designed (decrease in diameter of the dilator from 23.5 to 18 Fr.) and further used in stage-2. Primary outcome measure was immediate VP insertion without requiring additional instruments. Secondary outcome measures for comparison of the new with the traditional TEP procedure were: appreciation, ease of use, time consumption, estimated surgical risks and overall preference. A mini-max two-stage study design was used to establish the required sample size. In stage-1, dilatation forces were considered too high in patients with a fibrotic TE wall. With the final thinner version of the PVPS, VPs were successfully inserted into the TEP in ‘one-go’ in 24/27 (89%) of TEPs: 20 primary and 7 secondary. Participating surgeons rated appreciation, ease of use, time consumption and estimated surgical risks as better. Related adverse events were few and minor. The new PVPS appeared to be the preferred device by all participating surgeons. This study shows that the novel, disposable PVPS is a useful TEP instrument allowing quick and easy insertion of the VP in the vast majority of cases without requiring additional instruments.

## Introduction

Tracheoesophageal puncture (TEP) with immediate or delayed implantation of a voice prosthesis (VP) presently is the method of choice for restoring oral communication after total laryngectomy (TLE) [1]. A VP is a one-way valve, intended to prevent aspiration and to allow passage of pulmonary air into the esophagus, which initiates mucosal vibrations in the pharyngoesophageal segment and thus sound [2]. This sound subsequently is further processed to intelligible speech in the vocal tract.

The surgical procedure of VP implantation concerns a straightforward TEP procedure, either primarily during TLE or secondarily at a later stage. Secondary TEP was introduced for the first commercially available VP, the non-indwelling ‘duckbill’ prosthesis, by Singer and Blom [3]. In a series of 60 patients, these authors used a 14-gauge intravenous catheter set to puncture the TE wall into the lumen of a rigid esophagoscope and then used this intravenous catheter to pull a dilating 14 Fr. red-rubber catheter into the TEP. This second catheter was left in place for 7–10 days to stabilize the TEP allowing a 16 Fr. VP to be inserted and further maintained. This delayed insertion procedure was necessary because the original duckbill prosthesis did not contain an esophageal retention flange and the retention flanges of the successor non-indwelling devices were too thin to permit immediate insertion into the TEP. This was different for the first indwelling device developed in 1980, i.e., the ‘Groningen button’, which from the start was intended to be an indwelling device, immediately inserted at the TEP procedure, and therefore had a stable esophageal and tracheal flange [4]. In the first paper describing this indwelling device, immediate insertion without stenting of the TEP appeared to be feasible in 19 patients during a primary procedure, and in 14 during a secondary procedure [4].

Since then, primary TEP and immediate insertion of an indwelling VP have been the preferred methods in many European centers, also for other indwelling VPs developed later, such as the Provox and the Nijdam prostheses [5–7]. The main reason for this preference is that immediate prosthesis insertion eliminates the need for temporary stenting of the TEP with a catheter and allows for the earliest possible rehabilitation without the need for complicated prosthesis sizing and fitting at a later stage. At this stage, 10–12 days postoperatively, the stoma site is still sore, the TEP has not fully epithelialized yet, and the patients hardly have recovered from the procedure. The psychological boost of mostly instantaneous voicing, instead of voicing after a cumbersome sizing and fitting procedure, cannot be underestimated. Considering the relative ease of immediate VP insertion in the TEP while the patient is under general anesthesia, there is actually no real valid argument anymore to not immediately insert an indwelling device. Furthermore, the low complication rate of this primary insertion has been established in many clinical studies [7–10]. Also for the Blom–Singer indwelling devices, the feasibility and advantages of this approach recently have been recognized [11, 12].

Until present, the actual insertion of the indwelling device after the TEP was mostly achieved by ‘delivering’ the tracheal flange by pulling and rotating the VP into the TEP with the help of two hemostats. Although this procedure is not very complicated, it remains a delicate technique, which requires some force and time (somewhere between 2 and 4 min) in which the patient is not ventilated. Moreover, most of the current indwelling devices, such as the recently developed third generation Provox Vega prostheses, are distributed with the necessary instruments for anterograde replacement [13, 14], whereas immediate insertion during TEP requires the availability of a separate (dedicated or custom-made) guidewire. Therefore, a dedicated set for primary and secondary VP insertion is preferable. Moreover, the TEP procedure itself mostly is carried out with a special (Provox) trocar and cannula, or another surgical instrument, which need sterilizing and regular sharpening [5]. Therefore, a fully disposable set for immediate (primary or secondary) VP insertion, eliminating the need for additional instruments, could be helpful to further facilitate this procedure. Since Seldinger techniques nowadays are often used to achieve the introduction of medical devices by progressively dilating narrow puncture tracts, e.g., tracheotomy tubes [15], using such a technique would be an obvious choice. This paper describes the development and subsequent clinical assessment of such a TEP and immediate VP insertion tool in a prospective clinical multicenter trial.

## Materials, methods and patients

**Fig. 1:**
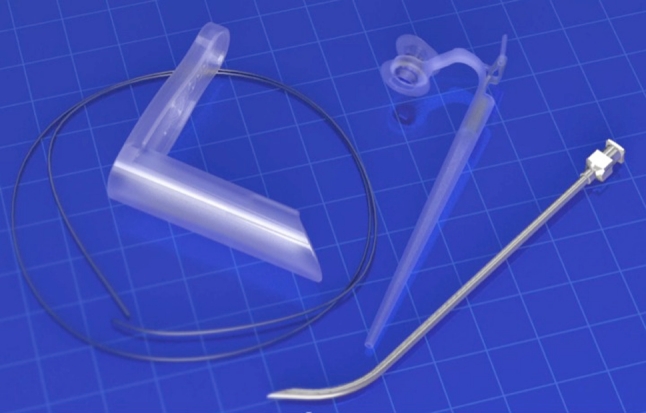
Overview of the disposable Provox Vega Puncture Set; *left* the pharynx protector (only to be used during primary TEP) and the guidewire; *right* the dilator (maximum diameter 18 Fr.) with the pre-loaded Provox Vega voice prosthesis and the puncture needle with a diameter of 2.5 mm

The newly developed Provox Vega Puncture Set (PVPS), based on the Seldinger technique, is a fully disposable, sterile set of instruments for primary and secondary TEP and immediate VP insertion. The set consists of a curved puncture needle to create the TEP, and a guidewire and a dilator with a pre-mounted Provox Vega VP for the dilation of the TEP and the actual introduction of the VP (see Fig. 1). There is also a basic plastic pharynx protector added, only to be used for primary TEP during TLE.

**Fig. 2:**
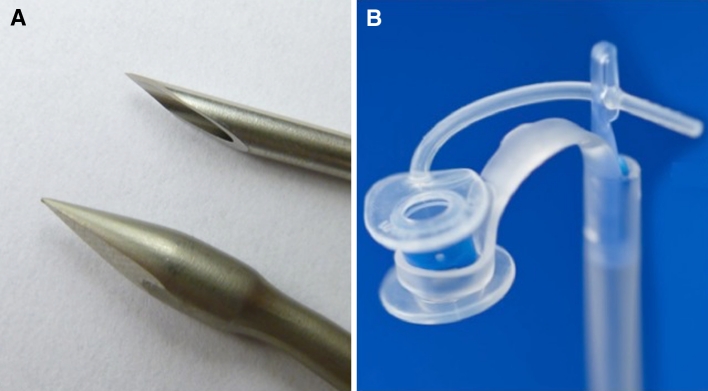
**a** Details of the new puncture needle (on *top*) with a maximum diameter of 2.5 mm and the trocar part of the Provox trocar and cannula (maximum diameter 5 mm); note the clear difference in outer diameter and sharpness. **b** Details of the dilator with the pre-loaded voice prosthesis and the special silicone loop around the shaft of the VP for the delivery of the tracheal flange of the voice prosthesis through the TEP tract

The first difference with the present Provox TEP and insertion method is that the needle is much sharper and thinner (diameter 2.5 mm) than the tip and diameter (in total 5 mm) of the Provox trocar and cannula mostly used now. Furthermore, instead of hemostats currently used for delivering the tracheal flange through the TEP, a dilator is used to progressively dilate the TEP tract and to deliver the tracheal flange through this tract into the tracheal lumen by means of a special loop around the shaft of the VP. Details of the needle and the loop are shown in Fig. 2.

**Fig. 3:**
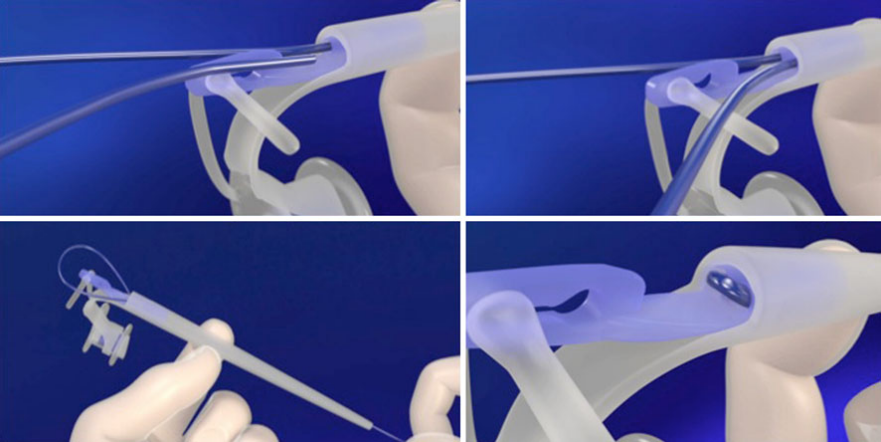
Details of the use of the guidewire lock: the guidewire is passed through the central lumen of the dilator (*top-left*) and the end of the wire is placed in the opening next to this lumen (*top-right*); next, the guidewire is pulled back (*bottom-left*) and securely locked into the dilator (*bottom-right*)

**Fig. 4:**
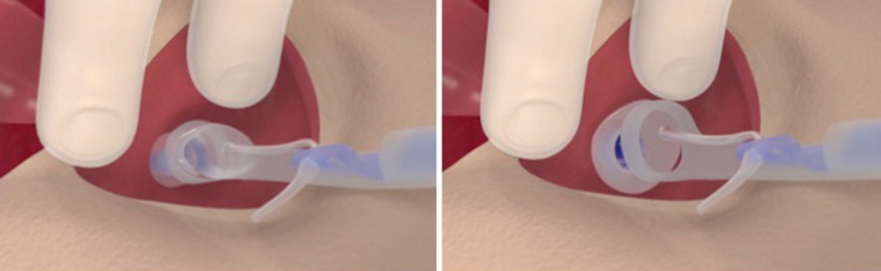
Function of the introduction loop of the dilator. During the passage of the voice prosthesis through the TEP tract, the loop pulls the tracheal flange forward and delivers and unfolds it into the trachea when it is ‘stripped off’ from the prosthesis shaft

In the PVPS, there is a guidewire, which is introduced into the TEP through the puncture needle with the pharynx protector still in place. After removal of the puncture needle and the pharynx protector, the guidewire is attached to the dilator with a simple locking system, as shown in detail in Fig. 3. The dilator itself is tapered with an increase in diameter from 3 to 18 Fr., which enables progressive dilatation of the TEP (the Seldinger principle). For the subsequent introduction of the VP into the TEP, the silicone loop at the end of the dilator folds the tracheal flange forward due to the traction on the dilator. During the subsequent narrow passage through the TEP, this loop slides over the shaft of the VP and over the tracheal flange, delivering and unfolding this flange out of the TEP into the tracheal lumen (see Fig. 4).

### PVPS development

#### Pre-clinical testing

The original diameter of the dilator of the PVPS was 25.5 Fr., based on the assumption that such a diameter was required for uneventful delivering of the tracheal flange of the VP, which has an outer shaft diameter of 22.5 Fr. After testing in fresh frozen cadavers, this diameter turned out to be too large considering the forces needed to dilate the TEP. Therefore, a smaller diameter (23.5 Fr.) was trialed, and with that version the forces needed for dilatation in a fresh frozen cadaver appeared to be more than halved. Therefore, this version was deemed suitable for clinical testing. After the inclusion of 20 patients (see below), the decision was taken to suspend the clinical investigation, since in two patients with a very fibrotic TE party wall [post (chemo)-radiotherapy], the dilatation forces were deemed unacceptably high by the surgeon. Although the primary outcome measure (successful insertion of the VP ‘in one-go’, see below) was still met in these procedures, it was decided to develop and trial smaller diameter versions of the dilator, to reduce the dilatation resistance even further. In subsequent cadaver testing with an 18 Fr. version, the forces needed to adequately dilate the TEP appeared to be reduced sufficiently, whereas immediate insertion of the VP still was feasible. Therefore, this 18 Fr. diameter dilator was deemed appropriate and the clinical investigation was restarted with this design (referred to as Stage 2 of this trial).

#### Study design

Once pre-operatively the indication for primary or secondary prosthetic voice rehabilitation was established, patients were informed about the use of the new TEP tool for VP insertion. The institutional review board (IRB) of all participating centers approved the study and a written informed consent was obtained from all patients.

Prior to the application, all surgeons were instructed about the proper use and order of handling of the PVPS, and had the possibility to practice the procedure in a dedicated tabletop model. Procedures were recorded on video for later re-evaluation and/or timing of the TEP with the PVPS. Immediately after completion of the procedure, data collection consisted of a study-specific questionnaire assessing each step of the procedure, registering possible complications and deviations of the methodology (i.e., the use of additional instruments such as hemostats), and overall physician satisfaction and preference. The structured questions could be answered either on a four-point ‘scale’ (e.g., on the question “Did you have any difficulties dilating the TE puncture?”, the possible answers were: 1 = not at all; 2 = a little bit; 3 = quite a bit; 4 = very much), or on a five-point ‘scale’ (e.g., on the question “Is the force needed to create the puncture with the needle lower or higher than the force needed to create the puncture with the Provox trocar and cannula?”, the possible answers were: 1 = much lower; 2 = slightly lower; 3 = similar; 4 = slightly higher; 5 = much higher). Surgeons were also asked to estimate the time they would have needed for the traditional Provox trocar and cannula method. Four weeks after the TEP procedure, an off-study form was completed, including registration of any complications. All adverse events and device effects were recorded and reported to the IRB(s).

#### Patients

As mentioned before, a multicenter clinical assessment of the PVPS was carried out in two stages. In the first stage, 20 patients were included, 16 males and 4 females with a mean age of 64.7 years (SD = 11.9; range 46–90). Nine patients underwent primary TEP with immediate VP insertion during the TLE, while 11 patients underwent a secondary TEP procedure with the PVPS. In 16 patients, an 8-mm Provox Vega was inserted, in 3 patients a 10-mm, and in one a 12.5-mm Provox Vega. All procedures were carried out under general anesthesia. In ten patients, the indication for TLE was recurrent disease after radiotherapy, of whom five had received chemoradiotherapy, five patients had postoperative radiotherapy and five were not radiated.

In stage 2 (using the final and actual design of the PVPS), 23 male and four female patients were included in the study, with a mean age of 62.0 years (SD = 7.8; range 46–78). Twenty patients underwent primary and seven secondary TEP with the 18 Fr. PVPS. The length of the VP applied was 8 mm in 24 patients and 10 mm in three patients. All procedures were carried out under general anesthesia. In eight patients, the indication for TLE was recurrent disease after radiotherapy, of whom four had chemoradiotherapy. Ten patients had postoperative radiotherapy, of whom two had chemoradiotherapy; nine patients were not radiated.

In all patients, the length of the prosthesis to be inserted was established by palpation of the thickness of the TE wall, either bi-digitally during primary TEP, or by palpating the TE wall onto the intraluminal device (mostly a rigid esophagoscope) during secondary TEP. All surgeons involved had extensive experience with this in the traditional Provox trocar and cannula method. Patients were entered in each center consecutively and, since in some centers the protocol review process required more time than expected, there were differences in contributions in the two stages of the trial. Patient characteristics and center contributions are provided in Table 1.

**Table 1:** Patient characteristics

	Phase 1 (*N* = 20)	Phase 2 (*N* = 27)^a^
Patients		
Males	16	23
Females	4	4
Center		
Amsterdam	6	5
Rotterdam	7	–
Ulm	7	13
Leuven	–	7
Barcelona	–	2
Timing		
Primary TEP	9	20
Secondary TEP	11	7

^a^‘Stop–go’ assessment: *N* = 23 (20 insertions correct without additional instruments, 85%); go: *N* = 4 (all correct in one-go); two additionally included patients had to be excluded because of protocol violation

#### Outcome measures

The primary outcome measure was the immediate insertion of the VP without the need of any additional instruments. Secondary outcome measures were: appreciation of the PVPS, ease of use of the new instrument set and procedure, time needed for insertion in comparison to the estimated time for the traditional Provox trocar and cannula method, the estimated surgical risk using the PVPS and the overall preference for the new PVPS or the traditional method.

#### Statistics

To introduce the device safely, a two-stage design, allowing for early stopping, with the following statistical considerations was applied: calculation of the number of subjects needed was based on an optimal mini-max design [16]. If the successes for the primary outcome measure is less than or equal to 70% (π_0_=0.7), the device is considered not (yet) suitable for implementation in clinical practice. If the percentage of successful insertions is 90% or more (π_new_ = 0.9), it can be concluded that the device is sufficiently safe to use. Furthermore, α is set to 0.05 and 1-β (power) to 0.8. With the parameters set accordingly, the sample size for the first stage (n_S1_; ‘stop–go’ point) is 23 and at least 20 successful insertions need to be observed to proceed to the next stage. If less or equal to 19 successes are observed, the trial should stop due to failure of the device. For the second stage, three additional patients have to be enrolled. Overall, a minimum of 22/26 successes will exclude with 95% certainty a success rate of 70% or worse and thereby indicate that the device is successful.

Statistical analyses of the data are mainly descriptive and include frequency tabulations, percentages and duration of the procedure. When variables are compared, a Wilcoxon signed ranks test is used. A *P* value of <0.05 is used to indicate statistical significance.

## Results

### Prior to redesign

In the first stage, 18/20 of the PVPS procedures (90%) resulted in immediate insertion of the VP, with only two procedures requiring the additional use of two hemostats. The mean recorded time needed for the new procedure was shorter (110 s; SD = 8; range 60–224) than that estimated for the Provox trocar and cannula method (201 s; SD 66; range 90–360), a statistically significant difference (*P* < 0.0001).

### Actual design of PVPS

In the second stage up to the ‘stop–go’ point, 20/23 PVPS procedures were successful ‘in one-go’ (87%) and finally 24/27 resulted in immediate insertion of the VP (89%). In two cases, two hemostats were used as additional instruments to insert the VP to completely deliver the tracheal flange, and in one case three hemostats. The mean recorded time needed for the trial procedure in the second stage was shorter (155 s; SD 116; range 58–600) than that estimated for the Provox trocar and cannula method (221 s; SD 58; range 120–330), also a statistically significant difference (*P* = 0.002). Mean insertion times for stages 1 and 2 were not different (*P* = 0.115). In stage 2, there were two outliers with 600 and 360 s, because of an unsuspected stenosis of the pharynx, which required dilatation first. Without those outliers, the mean insertion time in stage 2 was 110 s with a range of 58–224 s, and the mean insertion times for stage 1 and 2 were again not significantly different (*P* = 0.268). In stage 2, there were no cases anymore in which the surgeons considered the dilatation force unacceptably high, the reason for the re-design in stage 1. In Table 2, the main results are listed for both stages of the study.

**Table 2:** Primary and secondary outcome parameter(s) for the evaluation of the Provox Vega Puncture Set (PVPS) in comparison with the traditional Provox trocar and cannula method (PTCM); + favoring PVPS; = PVPS equals PTCM; − favoring PTCM; < easier/less time/less risk for PVPS; > less easy/more time/risk for PVPS

	Stage 1 (*N* = 20)	Stage 2 (*N* = 27)
Primary outcome measure		
Insertion in ‘one-go’	18 (90%)	24 (89%)
Extra instruments	18 none / 2 + 2 hemostats	24 none / 2 + 2 hemostats / 1 + 3 hemostats
Secondary outcome measures		
Mean time PVPS^a^	110 s (SD = 48; range 60–224)	155 s (SD = 116; range 58–600)^b^
Mean estimate PTCM	201 s (SD = 66; range 90–360); *P* ≤ 0.0001	221 s (SD = 58; range 120–330); *P* = 0.002
Appreciation	18 + / 1 =/ 1 −	25 + / 2 −
Ease of use	17 < / 2 =/ 1 >	26 < / 1 >
Time needed	16 < / 3 =/ 1 >	23 < / 3 =/1 >
Risk	14 < / 5 =/ 1 missing	20 < / 7 =
Preference	17 + / 3 −	26 + / 1 −

^a^Mean PVPS time for stage 1 and 2 is not significantly different (*P* = 0.115)

^b^There were two outliers with 600 and 360 s, because of an unsuspected stenosis of the pharynx, which required dilatation first. Without those outliers, the mean PVPS time was 110 s with a range of 58–224 s. Mean PVPS time for stage 1 and 2 is again not significantly different (*P* = 0.268)

As can be seen in Table 2 as well, in both stages the surgeons had a high appreciation for the new method and considered it easier to use than the traditional trocar and cannula method. Also, the overall risk of the PVPS was considered less in the majority of the procedures, leading to an overall preference for the new TEP tool. The length of the VP was judged correctly except in one case, where an 8-mm VP was implanted, which postoperatively needed re-adjustment (see adverse events).

Assessment of the technical aspects of the new procedure, i.e., the introduction of the pharynx protector in case of primary TEP, the actual TEP with the disposable needle, the introduction of the guidewire through the needle, the subsequent removal of the needle and the pharynx protector, the passage of the guidewire through the dilator and locking that into the slot, and the final dilatation and the actual introduction of the VP were considered trouble free in the majority of cases. In stage 2, the PVPS was used right-handed in 25 and left-handed in two cases, and all surgeons considered the new tool suitable for both approaches.

There were 27 instead of 26 patients included in the trial, because at the end of the study simultaneous informed consent was obtained from two patients on the same day (one in Ulm and one in Barcelona).

### Procedural inconsistencies

Inconsistent use of (elements of) the PVPS was rare. There were two (2) cases of retrograde insertion of the guidewire through the exposed tip of the needle, because the dilator already had been attached. In another case, the pharynx protector was not removed prior to attachment of the dilator, which was corrected by releasing the lock of the guidewire, removal of the pharynx protector and reattachment of the dilator with subsequent uneventful insertion of the VP. This latter error occurred in one other case (primary TEP), but then the surgeon pulled the VP through the pharynx protector, through which maneuver the VP got detached from the loop unnoticed, making the insertion with the PVPS impossible. This case was considered a protocol violation. The TEP nevertheless could be completed using the traditional two hemostats to ‘deliver’ the tracheal flange. A second protocol violation occurred in a patient, where the surgeon indicated needing a PVPS with a pre-loaded 10-mm VP, but when this appeared not to be available anymore, still tried to insert an 8-mm device. This 8-mm VP turned out to be too short for the tracheal flange to be delivered through the TEP by the loop. This case was solved using a regular 10-mm VP with the traditional Provox trocar and cannula method.

### Adverse events

In stage 1, there were four adverse events reported during the observation period of 4 weeks, three of which were deemed not to be related to the TEP procedure by the responsible surgeon. These three included two primary TEP patients developing postoperative wound infections outside the TEP area, requiring pectoralis major flap reconstruction, and one patient with pre-existing COPD developing postoperative pneumonia, responding to conservative/antibiotic treatment. The fourth case concerned a secondary TEP patient, who developed a peri-prosthetic leakage 2–3 days postoperatively, resolving spontaneously within a few days. This could be attributed to the wider dilator and the fibrosis present.

In stage 2, adverse events were reported in 8/27 patients, most of which were deemed not to be related to the TEP procedure. One patient developed tracheitis that responded to antibiotic treatment. In a secondary TEP patient, a mucosal tearing occurred in the stenotic pharyngeal wall during the rigid endoscopy procedure, but this did not interfere with the subsequent PVPS use. Three primary TEP patients developed postoperative fistulas, and one a neck abscess, all resolving by conservative measures. There were two adverse events considered to be related to the procedure, i.e., one case in which the prosthesis was too short and retracted partly into the TEP tract. Subsequent repositioning, however, solved this issue. In the other one, a secondary TEP case, the guidewire was passed outside the endoluminal device, penetrating the posterior pharyngeal wall. However, after discovering this, the guidewire was pulled back and still could be passed through the scope correctly and the PVPS procedure could be completed as intended. The patient had already received prophylactic antibiotics, as usual, and no problems developed postoperatively.

## Discussion

This development and subsequent multicenter clinical feasibility study shows that the novel surgical tool for primary and secondary TEP and immediate VP insertion based on the Seldinger technique, PVPS, is a versatile and easy to use instrument set, allowing immediate insertion of the VP in some 90% of the cases without the need of additional instruments. Although the estimated time gain is not very long in relation to the total OR time, this difference can be relevant, as during the TEP procedure, the patient is not ventilated.

With respect to a possible learning curve, if any, it is interesting to discuss in some more detail the few errors that were observed. In two cases, the instrument was not used appropriately. In one primary TEP, the pharynx protector was removed before introducing the guidewire, and in a secondary TEP the guidewire was not caught in the intraluminal device. As this guidewire is quite thin, it perforated the posterior esophageal wall. Fortunately, this had no clinical consequences, since the instruments used were sterile and the patient received standard prophylactic antibiotics.

With respect to the palpation of the TE party wall, in order to choose the correct VP length, there were very few mistakes. In fact only in one case a too short prosthesis was chosen, as mentioned under adverse events. This could be corrected by readjusting the tracheal flange and did not lead to early replacement of the VP. It has to be reemphasized that all surgeons participating in this trial had extensive experience with the method of TEP and immediate VP insertion, and this is certainly an important reason why in almost all cases the optimal VP length was used. For surgeons with less experience with TEP and immediate VP insertion, this might be different, although from experience with the traditional Provox TEP method, it is known that the learning curve is short as well. The reason is that in the vast majority of cases, the first VP used does not need to be longer than 8 mm (in this trial 39/47, 83%), and that only in a minority of cases a 10-mm version (7/47, 15%) is required, and exceptionally a 12.5-mm version (1/47, 2%) [5, 7]. The latter two lengths are mainly needed in secondary TEP cases.

In the literature, several TEP techniques have been described, all using some sort of a guidewire, either to establish the TEP tract, and/or to immediately insert the VP. Sometimes, this concerns the use of clinically readily available tubes, such as the red rubber catheter originally used by Singer and Blom [3]. Other times, this concerns dedicated instruments, such as the Groningen TEP trocar and flexible metal guidewire with a screw tip to perform the TEP and to attach and insert the VP [4]. Also, the original Provox disposable guidewire is a dedicated device, further simplifying the procedure [5]. More recently, Deschler et al. [11, 12] demonstrated the usability and versatility of this technique of primary TEP and immediate insertion of the indwelling Blom–Singer VP, by placing the VP on the tip of a 16 Fr. catheter and securing its strap with a silk suture to the catheter. These latter two papers show that primary VP insertion, which is a common practice Europe, is gaining popularity in the USA as well.

In conclusion, this prospective multicenter clinical feasibility study in 47 patients shows that this novel disposable tool for primary and secondary TEP and immediate VP insertion based on the Seldinger technique, the PVPS, is a useful instrument. It allows for immediate insertion of the VP in almost 90% of the cases without requiring additional instruments. The study showed a high degree of satisfaction with the PVPS and a substantial preference over the traditional Provox trocar and cannula method by the participating surgeons. This means that the PVPS can be considered an interesting addition to the armamentarium of surgeons already applying primary and secondary TEP with immediate VP insertion, but also that the PVPS can lower the threshold for those surgeons, who still delay the VP insertion after stenting the TEP tract with a catheter.
